# Relation between vitamin D deficiency and benign paroxysmal positional vertigo

**DOI:** 10.1038/s41598-021-96445-x

**Published:** 2021-08-19

**Authors:** Aida Ahmed Abdelmaksoud, Dalia Fahim Mohammed Fahim, Shamardan Ezzeldin Sayed Bazeed, Mohamed Farouk Alemam, Zaki Farouk Aref

**Affiliations:** 1 ENT Department, Faculty of Medicine, South Valley University, Qena, Egypt; 2 Audiovestibular Medicine (ENT) Department, Faculty of Medicine, Elminia University, Elminia, Egypt; 3 Tropical Medicine and Gastroenterology Department, Faculty of Medicine, South Valley University, Qena, Egypt; 4 Clinical Pathology and Clinical Chemistry Department, South Valley University, Qena, Egypt

**Keywords:** Diseases, Medical research

## Abstract

Benign paroxysmal positional vertigo (BPPV) is the most common cause of positional vertigo. Vitamin D deficiency may be one of the causes of its development. To assess the relation between recurrent attacks BPPV and Vitamin D deficiency. A case control study in which 40 patients were clinically diagnosed as posterior canal BPPV, Serum 25(OH) D was measured at 1st visit. Patients were divided into two groups; group A (20 patients) received Vitamin D supplementation in addition to canal repositioning maneuver and group B (20 patients) treated by canal repositioning maneuver only. Follow up of all patients for 6 months, neuro-otological assessment was repeated and recurrent attacks were recorded. Serum vitamin D was repeated after 6 month. This study included 14 males and 26 females age ranged from 35 to 61 years, Average serum of 25 (OH) D at the first visit was (12.4 ± 2 ng/ml) for group A, and (12.2 ± 1.7 ng/ml) for group B, all patients had low serum level of 25(OH) D (below 20 ng/ml). Recurrent BPPV episodes, were significantly lower in group A than that of group B. There is a relation between BPPV recurrence and low serum Vitamin D.

## Introduction

Positional vertigo is the vertigo induced by head motion^1^. Dizziness (including vertigo) affects about 15 to 20% of adults and vestibular vertigo accounts about a quarter of dizziness complaints^2^. BPPV is defined as recurrent positional vertigo attacks^1^ and is considered as the commonest vestibular disease^3–5^ its incidence rate among population is approximately 10%^6^. It is more common around age of sixty^7^. Dislodgement of calcium carbonate crystals (otoconia) from the utricle into the semicircular canals (most commonly the posterior canal) is one of the accepted theories of pathogenesis of BPPV. Vitamin D plays a major role in Calcium metabolism which may affect the calcium carbonate crystals (otoconia) density and matrix^8^.

The otoconia crystals consist of two parts; central core and peripheral zone. The core is mainly organic (which is predominantly glycoprotein) with a lower level of Ca^2+^, and the peripheral zone is mainly inorganic (which is predominantly a polymorph of calcium carbonate CaCO_3_) with a higher level of Ca^2+^^9,10^. The core, periphery and external surface of the crystals all have inter-connecting fibrous material with varied diameters and organization. The crystals of otoconia are formed by the active calcium metabolic process of the vestibular organ. Otoconia crystals are partially embedded in a fibrous matrix and are connected to hair cells with protein fiber^11^.

Calcium absorption from intestine and kidney increased and affected by vitamin D. The maintenance of blood calcium is controlled by parathyroid hormone. Vitamin D has direct effect on the mechanism of deposition of calcium and phosphate in the bone, teeth and otoconial particle formation in the vestibular system^12^. There are common features between bone and otoconia biomineralization. As the matrix organization and protein constituents are similar between the two tissues. Biomineralization in otoconia involves tight regulation of the formation of an organic matrix at specific sites and the deposition of mineral crystallites in an ordered manner similar to that in bone and teeth^8,13,14^.

In osteoporosis, there is a disturbance in the metabolism of both vitamin D and calcium and this is probably the key element of the pathogenesis of BPPV. Vitamin D level and deposition of calcium crystals affect the otoconia matrix and density similar to its effect on bone structures^8^. Vitamin D insufficiency correlated with the severity of BPPV and its recurrence^15–17^. In fact, the recurrence attacks of BPPV may decreased with vitamin D supplementation. Interestingly, a large number of cases was accomplished a complete remission after trials of vitamin D supplementation^18,19^.

The association between BPPV and osteoporosis was postulated in some studies^20^. This relationship originate from the essential role of calcium metabolism in the homeostasis of otoconia metabolism which regulate the synthesis and absorption of otoconia, which mainly composed of calcium carbonate^21^. So that any disturbance in calcium metabolism, as in osteoporosis and osteopenia, may contribute to the development of BPPV^22^. BPPV was related to a 1.28 times higher odds of osteoporosis. In addition, osteoporosis was associated with a 1.34 times higher odds of BPPV^23^.

Vitamin D are maintained in its adequate level through its cutaneous photosynthesis and oral ingestion. By some estimates, one billion people worldwide have vitamin D deficiency or insufficiency. Photosynthesis and bioavailability of vitamin D influenced by many factors and these factors may contribute to risk of impaired vitamin D status. These factors include variation in sun exposure due to geographic latitude, time of day, solar radiation exposure, season, weather condition, air pollution, clothing, sunscreen use and skin pigmentation, as well as age, obesity and the incidence of several chronic illnesses^24^.

Although BPPV may result secondary to migraines, head trauma, vestibular neuritis, prolonged bed rest and otologic surgery, 80% of all cases are idiopathic^25^. Canal repositioning maneuver (CRM) is rapid and effective in which the dislodged crystals are returned to the utricle where they may be absorbed by the body. However, BPPV often recurs despite the effectiveness of this treatment. Many researches have confirmed that vitamin D receptors are founded on calcium channel transport systems of the labyrinth and act to regulate proper calcium balance. This mechanism may help to explain the role of vitamin D in maintaining proper auditory function^25^. Deficiency of vitamin D has been attributed to cochlear demineralization and cochlear deafness. The deficient vitamin D may exert its effect by disturbed calcium metabolism as calcium ions play an important role in membrane permeability. Ionized calcium is necessary for normal function of the nerve and its deficiency may affect the action potential generation in cochlea. Low level of vitamin D and calcium may lead to demineralization of otic capsule, degenerative changes in the spiral ligament, stria vascularis, and cochlear hair cells^26^. Brooks et al. has reported improvement in the degree of hearing after restoration of serum vitamin D level^27^.

## Aim of the work

The aim of this study is to assess Vitamin D serum level in BPPV patients and to assess the relation between recurrent attacks of BPPV and Vitamin D deficiency, Also to evaluate the effect of Vitamin D supplementation in decreasing number of recurrent attacks of BPPV.

## Patients and methods

### Ethics approval and consent to participate

The study was approved by Qena Faculty of Medicine Ethics Committee. The reference number of the committee is 54/4/7/2020. We confirm that all research was performed in accordance with relevant guidelines/regulations; informed consent was obtained from all participants and/or their legal guardians in accordance with the Declaration of Helsinki.

Verbal informed consent to participate in the study was obtained from parents or legal guardians of all cases. The consent was verbal as most of our cases parents or legal guardians were not educated. The Qena Faculty of Medicine Ethics Committee approved the verbal consent in our study.

Forty patients suffering from BPPV presented at the Qena university hospital and private clinic from January 2019 to June 2020. These patients were included in the study after obtaining written and oral consent. These patients include 26 female and 14 male their average age was 48 years.

#### Inclusion criteria

Diagnosis of canalithiaisis or cupulothiasis BPPV of posterior semicircular canal which was made based on history (Recurrent episodes of positional vertigo or positional dizziness induced by turning over or lying down in the supine position and the attacks ends < 1 min), clinical examination (Positional nystagmus appears after a latency period of few seconds in canalithiaisis or elicited after a brief or no latency in cupulothiasis by the Dix-Hallpike maneuver. The nystagmus is a mix of torsional nystagmus with the upper pole of the eyes beating toward the lower ear combined with vertical nystagmus beating upward typically lasting < 1 min in canalithiaisis and > 1 min in cupulothiasis and not caused by another disorder^28^.

#### Exclusion criteria

History of head and ear trauma, surgery or infectious disease of the ear during the preceding month of BPPV attack to exclude secondary BPPV. Also patients with chronic renal diseases, pulmonary, hematologic, gastrointestinal and cardiovascular diseases. Also patients taking Supplementary calcium and/or Vitamin D or taking medication that alter Vitamin D metabolism in the last year, Patients with history or active case of inner ear disease. Patients with a typical history of BPPV without exhibit nystagmus on clinical tests and Patients whose data is incomplete or denied for consent may be excluded^29^.

Patients were subjected to a detailed history taking, clinical examination for spontaneous nystagmus, headshaking nystagmus, positional and positioning nystagmus using frenzel google and laboratory investigation of Vitamin D by measuring serum 25 hydroxy Vitamin D level at first visit. All patients should had history of previous visiting the clinic with the diagnosis of BPPV of at least 2 or more attacks of BPPV over 6 months prior to inclusion in the previous 2 years.

Vitamin D status was classified according to measured 25(OH) D concentration: less than 10 ng/mL: deficient; between 11 and 20: insufficient; higher than 20 ng/ml: optimal^30^ For patients with insufficiency and deficiency serum level and no history of nephrolithiasis, Vitamin D supplement was given in regimen of cholecalciferol 8000 IU daily for 2 weeks followed by 4000 IU daily for 2 weeks then 8000 IU single dose weekly for 3 months^31^.

CRM was done for all patients at the first visit once and the patients were instructed to visit the clinic 2–3 days later. The disappearance of symptoms and disappearance of nystagmus in Dix-Hallpike test was considered as criteria for complete cure and successful maneuver. Instead occurrence of vertigo 1 month or more after complete recovery which led to identification of nystagmus with frenzel glasses and consequently to diagnose BPPV was considered as criteria for recurrence.

Patients were divided randomly into two groups group A (received supplementary Vitamin D and CRM was done to them) and group B (CRM only was done to them). Follow up period was 6 months. During this period the clinical assessment was repeated and serum Vitamin D level was repeated for both groups after 6 months. Patients assessed and reassessed at first visit, after 1 month, 2 months, 3 months and 6 months. The number of BPPV attacks in each group was recorded over 6 months follow up period in both groups.

### Statistical analysis

Data were analyzed using Statistical Program for Social Science (SPSS) version 24. Quantitative data were expressed as mean ± standard deviation (SD). Qualitative data were expressed as frequency and percentage. Mean (average): the central value of a discrete set of numbers, specifically the sum of values divided by the number of values. Standard deviation (SD): is the measure of dispersion of a set of values. A low SD indicates that the values tend to be close to the mean of the set, while a high SD indicate that the values are spread out over a wider range.

The following tests were done:

Independent-samples t-test of significance: was used when comparing between two means. Chi-square test: was used when comparing between non-parametric data. Probability (P-value) P-value < 0.05 was considered significant.

## Results

In this study 40 patients diagnosed with posterior canal BPPV were included. Successful epley maneuver was performed and serum Vitamin D levels were calculated in the first visit. Besides we select only patients with vitamin D less than 20 ng/ml.

Patients were divided randomly into two groups, group A with mean age (49.9 ± 7.4) and group B with mean age (50.2 ± 9.6) with no significant difference (Table 1). 20 patients in Group A; 6 males (30%) and 14 females (70%), also in group B: 20 patients were included; 8 males (40%) and 12 females (60%) with no significant difference (Table 1). As regard serum Vitamin D level of the first visit, group A mean serum level was (12.4 ± 2) versus (12.2 ± 1.7) for group B also with no significant difference between both groups (Table 1).

**Table 1:** Comparison between demographic data of both groups.

Variable	Vit D and epily (group A 20 patients)	Epily only (group B 20 patients)	P value
Age	49.9 ± 7.4 Average = 49.9	50.2 ± 9.6 Average = 50.2	0.9
**Sex**			
Males	6 (30%)	8 (40%)	0.6
Females	14 (70%)	12 (60%)	
Vitamin D level in the first visit	12.4 ± 2 Average = 12.44	12.2 ± 1.7 Average = 12.23	0.8

After 6 months of treatment and Vitamin D therapy in group A; Vitamin D serum levels were increased with mean level (26.3 ± 4.1) but in group B vitamin D serum level didn’t show any change with mean level (12.2 ± 1.7) with highly significant difference between group A and group B (Table 2). The mean value of recurrence in 6 months follow up duration was (0.2 ± 0.4) in group A in comparison to (1.5 ± 0.7) in group B with highly significant p value (Table 2).

**Table 2:** Comparison between vitamin D level and recurrence rate after 6 months in group A and group B.

Variable	Vit D and Eply	Eply only	P value
Vitamin D level after 6 months	26.3 ± 4.1	12.2 ± 1.7	0.000*
Recurrence during 6 months period	0.2 ± 0.4	1.5 ± 0.7	0.000*

∗highly significant.

In comparison between Vitamin D serum level 6 months of Vitamin D therapy there were significant increase in the mean serum Vitamin D level (26.3 ± 4.1) versus (12.4 ± 2) in the first visit with highly significant p value (Table 3).

**Table 3:** Comparison between vitamin D level in group A in pre and post vitamin D therapy.

	Vit D in groupA first visit	Vit D in group A pos V D therapy	T value	P value
Vitamin D	12.4 ± 2	26.3 ± 4.1	18.2	0.000

**Figure 1:**
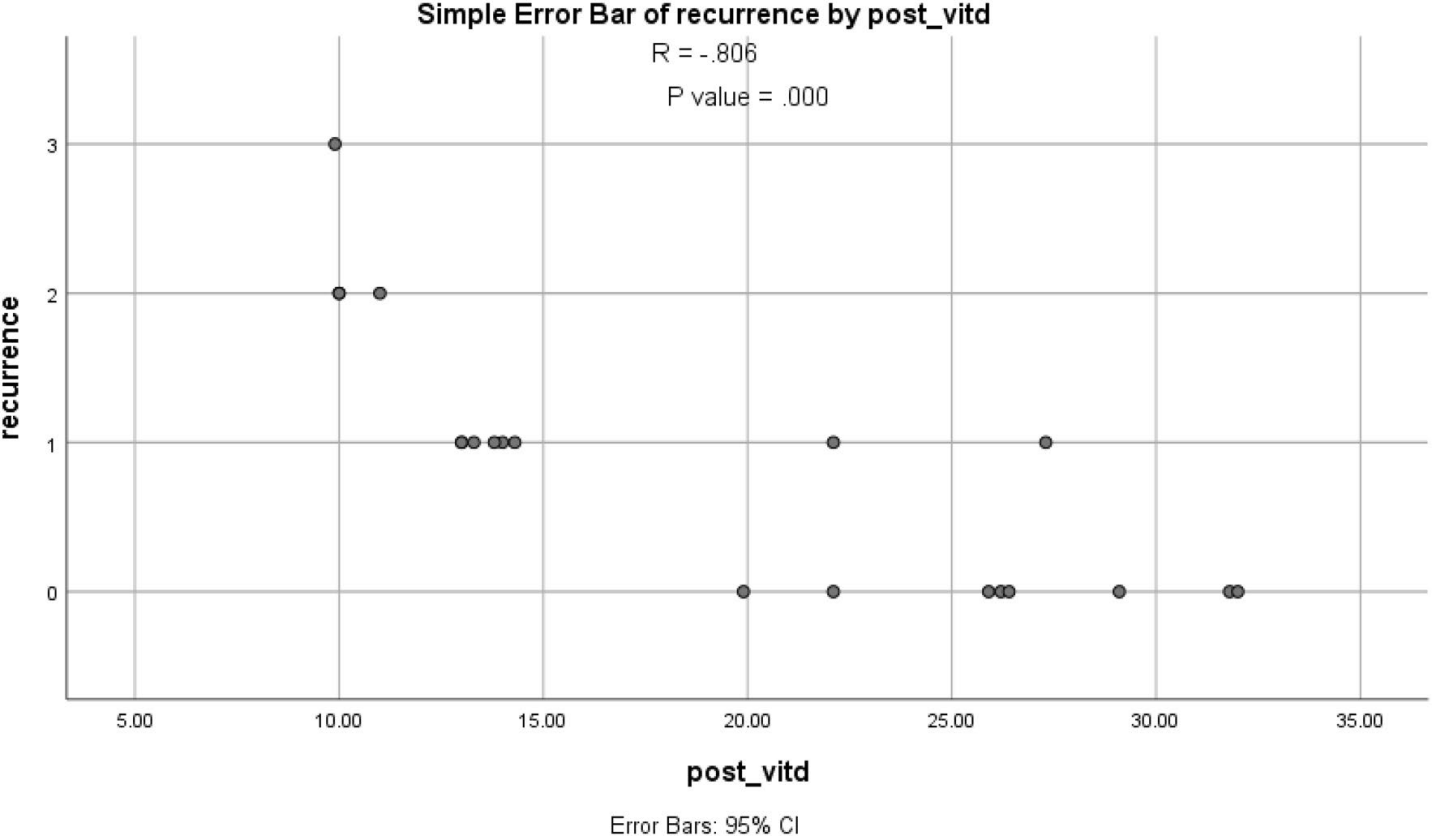
Correlation between Vitamin D and BPPV recurrence.

As regard correlation there were negative correlation between recurrence rate of BPPV episodes and Vitamin D deficiency with R = − 0.806; which means that Vitamin D deficiency may have a role in recurrence of BPPV (Table 4, Fig. 1).

**Table 4:** Correlation between VIT D and recurrence.

	R	P value
Vit D and recurrence	− 0.806	0.000*

∗highly significant.

The number of episodes during 6 months of follow up: in group A who received Vitamin D therapy in addition to Epley maneuver 4 patients out of 20 patients developed one episode in 6 months follow up duration, and the recurrence was near the 1 month in 4 patients but in group B who received Epley maneuver only: 12 patients (60%) developed one episode, 6 patients (30%) developed 2 episodes, and 2 patients (10%) developed three episode and the recurrence was in different times in the follow up month but almost of them had the 1st recurrence in 1st month. There were statistically significant difference between group A and group B as regard number of recurrent episodes with p value = 0.003 (Table 5).

**Table 5:** Comparison of number of recurrent episodes during 6 months follow up in group A and group B.

Recurrence in 6 months duration	Group A	Group B	P value
0 episode	16 (80%)	0 (0%)	0.003*
1 episode	4 (20%)	12 (60%)	
2 episode	0 (0%)	6 (30%)	
3 episodes	0 (0%)	2 (10%)	

∗highly significant.

## Discussion

Egyptian people have a high incidence of Vitamin D deficiency, which may be related to different factors influencing vitamin D as clothing, variation in sun exposure, age, obesity, several chronic illnesses also there is seasonal variation as vitamin D deficiency especially in season there is less exposure to sun light. A seasonal variation of patients presenting with BPPV has been observed in the United States and Iraq. In Boston, the number of BPPV clinic visits was greatest during March to May, months where serum vitamin D levels are at their lowest^16,32^. Populations living in sunny, neighboring regions of the Equator, have high serum vitamin D levels due to higher radiation incidence from the sun with fairly short wavelengths, ultraviolet B (UVB)^33^.

BPPV Patients were found to have low serum Vitamin D level less than20 ng/ml. This is almost near that of Austrian population who has average 20.9 mg/ml^17^. Patients in this study had significantly low serum level of 25(OH) D, Mean level of 25(OH) Din group A was (12.4 ± 2) and in group B was (12.2 ± 1.7). The number of women was higher in the study groups, but with no statistically significant difference. Which is similar results of Study done by Rhim, the number of women was higher in the study group (P = 0.051), but this difference was not statistically significant, and this difference is explained with differences in sex hormones^34^, another study done by Mithal et al. who reported that the incidence of Vitamin D deficiency was also more among females to^35^. The mean age of our study groups was 48 years which is also near to results of Rhim^34^ in which the median age of subjects was 50 years.

Our study showed that the mean of recurrent attacks of BPPV in group A (0.2 ± 0.4) was significantly lower than that in group B (1.5 ± 0.7). There was a negative correlation between recurrence rate of BPPV episodes and Vitamin D deficiency which means that Vitamin D deficiency may have a role in recurrence of BPPV. These results are similar to other studies. Recurrence rates of BPPV are known to be 30–50% between 3 and 5 years^36,37^, and usually associated with female sex, old age, ear diseases, lateral canal, chronic diseases, and Vitamin D deficiency^36–39^. Rahim et al. assessed the recurrence rates of BPPV episodes for a long period of time without limiting the age, sex or locations of semicircular canals, Patients had history of BPPV recurrent episodes before therapy 3–4 episodes/year. After Vitamin D therapy, there was significant decrease in the recurrence rate during 6 months of follow up and he concluded that serum Vitamin D concentrations significantly affect the recurrence of BPPV^34^.

Büki et al. found that his patients with idiopathic Benign Positional Vertigo had low Vitamin D serum levels (23 ng/ml)^16^. He recorded 4 patients having recurrent episodes of BPPV for a longer period before examination with a frequency of 4–6 episodes/year for several years. These patients had statistically lower serum Vitamin D level than patients with the first episode. After Vitamin D therapy, BPPV patients have not reported recurrent episodes in the follow up period for 8 months of follow up^16^.

In another meta-analysis that investigated the difference between the recurrence and non recurrence of BPPV, there was a significant difference in the Vitamin D levels between the two groups, which indicated that Vitamin D play, a role in the recurrent nature of BPPV. Consistently, it reported that the BPPV recurrence was frequent among the patients with osteoporosis^20^. Mohsin et al. reported also that patients with BPPV had low serum level of Vitamin D (11.6 ng/ml). He recorded 9 patients had recurrent attacks of BPPV for several years with a frequency of 3–4 relapses/year. These patients were found to have statistically lower serum level of Vitamin D than patients with first episode. After Vitamin D therapy patients with BPPV reported no episodes in 10 months follow up duration^6^.

In 2003 Vibert et al. found that the recurrence rate of BPPV was 27% and most patients relapse in the first 6 months. He also reported a relation between BPPV and osteoporosis, since bone metabolism may have a role in pathogenesis of BPPV^40^. Yamnaka et al. found that the incidence of osteoporosis in BPPV patients was 26.2% and the incidence of recurrent episodes of BPPV was 56.3%. These rates were statistically higher than that recorded in patients with normal bone density (16.1%)^41^. Jeong et al. found that the levels of serum 25(OH)D in 100 patients with idiopathic BPPV was lower than that in control group^42^, and the incidence of 25 (OH)D insufficiency (< 20 ng/ml) in patients with BPPV was significantly higher than that in control group. Xiang et al., proved that both the 25(OH)D insufficiency (10–20 ng/ml) and deficiency (< 10 ng/ml) are associated with BPPV, and suggest that low serum 25(OH)D may have a role in the pathogenesis of BPPV^1^. Xiang also reported that in cases with chronically recurrent severe BPPV episodes, low levels of serum 25 (OH) D could be measured and, BPPV did not recur after supplementation with Vitamin D^1^.

Hae and his colleges in 2013 found serum vitamin D lower in patients with BPPV than healthy controls. Patients with recurrent BPPV had lower Vitamin D serum than other patients. They recommended that serum Vitamin D should be above 30 ng/ml with safe sun exposure for up to 30 min per day to decrease recurrence of BPPV^43^. In another study vitamin D supplementation was given to patients with posterior semicircular canal BPPV and vitamin D deficiency for 3 months. In follow up of those patients the recurrence rate was significantly lower than patients did not receive Vitamin D supplementation which is similar to this study^18^.

## Conclusion

Our data suggest that most patients with BPPV in Egypt have low serum Vitamin D and Vitamin D supplement may have a role in decreasing recurrent attacks of BPPV. Further studies must be done to assess role of Vitamin D therapy in treatment of BPPV.

### Recommendation

Larger sample size will give more information about both vitamin D deficiency and BPPV and their relation.

### Limitation

Vestibular Migraine is highly associated with occurrence of BPPV and its a cause of positional vertigo which may confused with recurrent BPPV and it is also a possible cause of BPPV recurrence.

## Data Availability

The datasets used and/or analysed during the current study are available from the corresponding author on reasonable request.
